# Calf muscle perfusion maps from contrast-enhanced magnetic resonance imaging (CE-MRI) to assess peripheral arterial disease

**DOI:** 10.1186/1532-429X-16-S1-P170

**Published:** 2014-01-16

**Authors:** Gerd Brunner, Jean Bismuth, Vijay Nambi, Christie M Ballantyne, William Zoghbi, Alan Lumsden, Joel D Morrisett, Dipan J Shah

**Affiliations:** 1 Baylor College of Medicine, Houston, Texas, USA; 2 The Methodist DeBakey Heart & Vascular Center, Houston, Texas, USA; 3 Michael E DeBakey VA Medical Center, Houston, Texas, USA

## Background

Peripheral arterial disease (PAD) is associated with impaired function of the lower extremities, manifested clinically as intermittent claudication or rest pain. Previous studies have suggested that alterations in microcirculation due to a paucity of collateralization may contribute to functional impairment in PAD patients. Therefore, small vessel blood flow at the level of leg muscles may be important. We hypothesized that microvascular perfusion can be measured with CE-MRI and will be heterogeneous in calf muscles of PAD patients compared with healthy controls.

## Methods

Twenty six PAD patients and 14 healthy controls underwent CE-MRI with a 3.0T Siemens Magnetom Verio system using a 36-element dedicated peripheral angio-matrix coil. A state of reactive hyperemia was induced by 3.5-minutes of supra-systolic inflation of a bilateral blood-pressure cuff positioned above the knee. Rapid cuff deflation was synchronized with the administration of a Gadolinium-based contrast agent (Magnevist, Bayer Inc.). Imaging at the mid-calf was commenced with a high resolution saturation recovery gradient echo pulse sequence (repetition time [TR] = 2.7 ms; echo time [TE] = 1.23 ms; and temporal resolution = 409 ms per frame). Microvascular perfusion maps (MVPM) were calculated for each frame and for five semi-automatically segmented skeletal muscle compartments including the anterior, lateral, deep posterior, soleus, and gastrocnemius. The MVPM categorized each voxel as either hypointense (voxel signal intensity (SI) < reference SI - 2*standard deviation [STD]), isointense, or hyperintense (voxel SI > reference SI + 2*STD), based on reference signal intensities for each muscle group that were calculated from all controls using an ensemble means algorithm. Normalized histograms of MVPM representing fractions of hypo-, iso-, and hyperintense voxels were compared for each muscle group between PAD and controls. Analysis was performed for specified times based on the arterial input function (AIF) of one sufficiently patent major artery (anterior tibial artery, posterior tibial artery, or peroneal artery) including pre contrast arrival (CA), peak AIF, and 2 minutes post CA.

## Results

**Figure 1:**
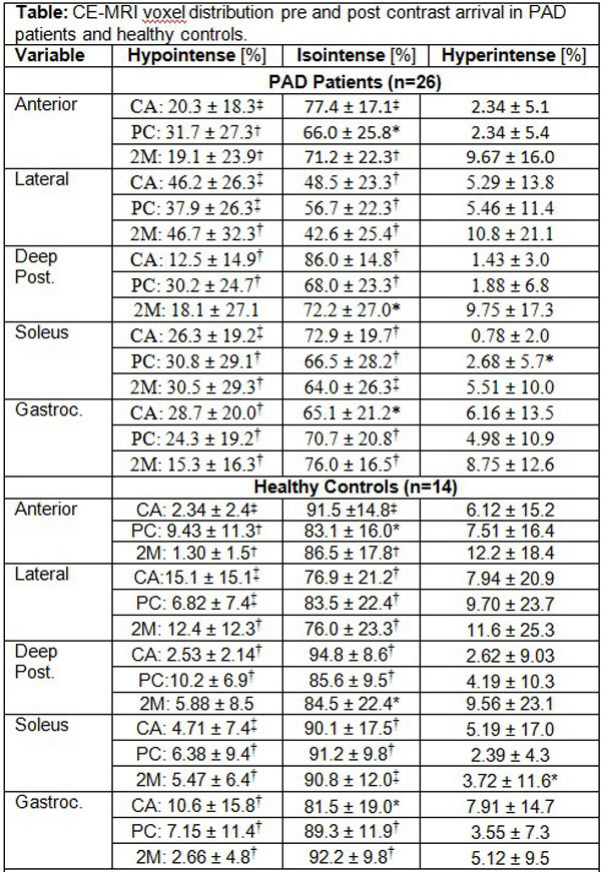
*p < 0.05; †p < 0.01; ‡p < 0.0001; Otherwise not significant. P-values for group differences were calculated with the Wilcoxon rank-sum test (SAS 9.3). CA: pre contrast arrival; PC: peak arterial enhancement; 2M: 2 minutes post contrast arrival.

**Figure 2:**
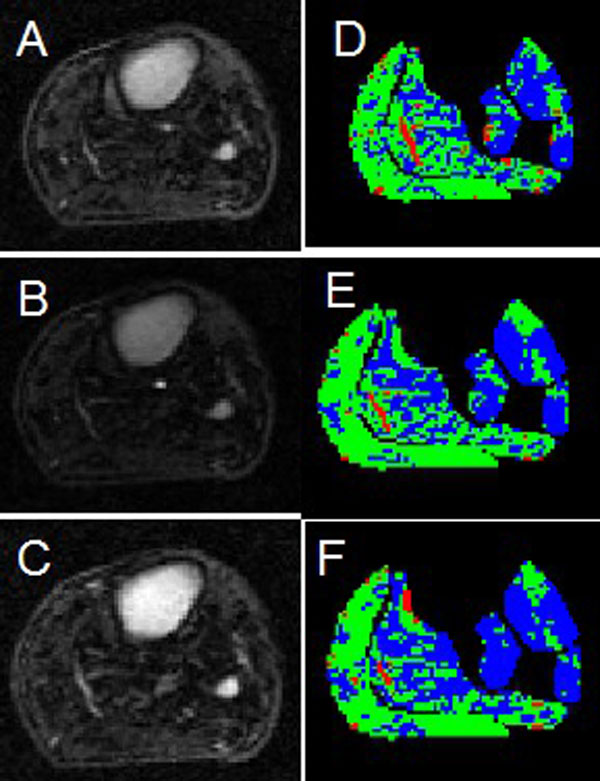
**CE-MRI images from a PAD patient (panels A-C) prior to contrast arrival (CA, panel A), at peak arterial contrast enhancement (panel B), and 2 minutes post CA**. Panels D-F show the respective perfusion maps where blue, green, and red colors indicate hypointense, isointense, and hyperintense regions, respectively.

The 26 PAD patients presented with an average ankle brachial index of 0.624 +/- 0.18. Only the more symptomatic leg was analyzed. The anterior, lateral, deep posterior muscle compartments and the soleus and gastrocnemius muscles presented with a higher fraction of hypointense voxels in PAD patients when compared with healthy controls at 3 distinct times during perfusion imaging (p < 0.01; Figure 1). PAD patients had a lower amount of isointense voxels for the five muscle compartments compared with controls (p < 0.05). However, there was no significant difference between the fraction of hyperintense voxels (Figure 2).

## Conclusions

The preliminary results of this study suggest that CE-MRI may be useful to assess microvascular perfusion abnormalities in patients with PAD.

## Funding

This work was supported in part by The Methodist DeBakey Heart & Vascular Center (MDHVC) Research Award 2011, the Society for Vascular Surgery (SVS): 2012 Clinical Research Seed Grant, and NIH grant T32HL07812.

